# Reflex Nervous Disorders from Dental Irritation

**Published:** 1876-03

**Authors:** 


### REFLEX NERVOUS DISORDERS FROM DENTAL IRRITATIO.N

*A paper was read on this subject by Dr. A. L. Carroll, for which see Jan. number of Register.*

The discussion upon the subject of the paper was opened by Dr. John M. Riggs, Hartford, Conn., he said that the paper brought to his mind many of the scenes he had witnessed in relation to the pains attendant upon diseased organs of the teeth. There was no doubt, that neuralgic pains in the head arise from the teeth. The teeth may be effected in different ways to produce neuralgic difficulties. The pain caused by the teeth is directly traceable to the reflex action of the nerves. The case that the doctor referred to in the morning, where the teeth were all extracted to cure neuralgia, was probably the case where all the teeth were diseased from the inflammation, suppuration of the gums and absorption of the alveolus; at least it looked to him like a case of that kind. That disease frequently caused partial blindness and also epileptic fits; he had seen in several cases, that extracting the teeth in such instances would cure the trouble at once; but only in cases when the teeth were the cause of the trouble. He had cases where the patient was so blind that he could not read print; he was perfectly cured by the extraction of the teeth and restoring the gums, and stopping the absorption of the alveolar process.

Dr. Abram Robertson, Georgetown, Mass., said that the paper read, fully corroborated his own observations and experience. The first paper of a professional kind that he had ever written (on this subject) he read in November, 1854, before the old Society of Dental Surgeons; showing the effects on the general system, that it was the direct cause through the nervous relation of the development of many diseases. He said he treated a patient and removed a diseased tooth, which had not only relieved neuralgia, but relieved the patient of rheumatism he had in his shoulder.

Dr. W. S. Elliott, Goshen, N. Y., said that when the physician was called upon to diagnose a tooth ache, and when he did not know the reflective action from irritation at some remote point, he would be entirely dissatisfied with the diagnosis. It is very easily understood that local irritation of the teeth or of the other organs, could manifest itself in remote parts of the system; but he would like to hear more on direct reflection.

Dr. E. D. Miles, Philadelphia, Pa., said that he doubted whether there would be any manifestation in perfect teeth of general derangements; the tooth must be imperfect. Where there is any difficulty in the periphery, this reflex action was probably quite manifest. There had been a theory that they could tell where the difficulty was by approaching the nerve center, that if there was a tender point any where they had an indication that the trouble should be at the terminus of that nerve. He did not believe where there was difficulty in the teeth from decay, but it could be mitigated by local treatment, and that these general difficulties, for instance, during gestation, lactation, cold or excitement, traumatic injuries would all subside by a simple treatment, and that the phenomena would cease if the teeth were in perfect condition. He presumed that all these influences were reciprocal and that the teeth affect the body in such a way as to find response where there was any difficulty whatever—and *vice versa.*

Dr. Geo. H. Perine, New York, said that the subject was one of exceedingly interesting nature, and worthy of long and serious debate. The question was one of those subjects which were almost endless as well as encouraging, as to their satisfactory solution. The doctor proposed that the subject be discussed now at length.

Dr. William H. Atkinson, New York, said: When it comes to differentiation between the two professions, as to whether I shall call myself a medical man or a dentist, I will stand with the dentists! But, lest the medical gentlemen present should not apprehend the warm affection I bear to all interested in medicine, I would like to explain. In an address I once delivered, I said that I for one, would require candidates for dentistry to be first A. B's., next M. D's., and then we would give them dental skill, and finally bring them to a proper institution to be trained in dental surgery. We can not be perfect, but we can advance towards perfection.

Dentistry has no reason to blush in the face of medicine. The army records show that the surgical operations of the dentists equaled those of the best surgeons. A pupil of mine who has passed "behind the curtain," once performed, in the army, the operation of amputation of the hip-joint; and so dexterously was it done that several of the surgeons came up and complimented him upon it. Said one; "That is not your miaden operation by any means!"—"What do you mean?"—"You have made that operation many times before; for there is no man living who could have made it without having gone over it again and again, upon the living body." He had never performed the operation upon the living body before.

Dr. Fred. S. Howard, New York, said: Dr. Atkinson's remarks come home to me in regard to the delicate skill which the dentist is called upon to exercise. Prof. Darling, of the Medical Department, University of New York, once told me that those students who had experience in the dental art, made the best surgeons that they graduated from their college. The delicate touch of the dentist comes by experience, and we recognize the touch in every operation we perform.

Dr. E. D. Miles, Philadelphia, said: The touch spoken of is a most radical accomplishment. Some men are unfitted by nature, to acquire it. How often do we hear the remark: "He is so rough! I will never go back to that dentist!" The very touch of the man is repugnant; and although he may have done his work well, he is disparaged because of his awkwardness.

Dr. John M. Riggs, Hartford Conn., said: The surgeon must be born a surgeon. A gross-minded man never can make a good surgeon. The motto of the poet applies here: *pœta nascitus non fit*. This is eminently true of the dentist. At the present day, we need the nicest manipulation. The dentist, as well as the surgeon, must have his eyesight at the ends of his fingers.

The next paper read was entitled "The Tooth Brush as a Prophylactic," by Abram Robertson, M. D., D. D. S., Georgetown, Mass. The usual discussion followed.

Dr. Fred. S. Howard said that in speaking of the tooth brush as a prophylactic, he supposed that the expression carried with it all the improvements and every thing that was necessary to one's personal wants. It was told in Knickerbocker's History of New York, that when the country was first populated, the Aborigines were found in such a deplorable condition that they could not appreciate the value of civilization, that they could not understand the value of medicine, for the reason that they had no disease that medicines were necessary to cure, and in order that they might appreciate the value of medicines, our forefathers introduced the diseases. So with the tooth brush, it was used, not the same as the ear-spoon (which instrument he thought was a barbarous instrument) but still used because it is fashionable. Only the exposed portions of the teeth are brushed by many people, the approximal surfaces left uncared for. He recommends not only the brush, the floss silk, the wood, but also the very best attention.

Dr. A. W. Kingsley, Elizabeth, New Jersey, desired to know if they were all to give up tooth brushes and advise their patients not to use them. He brought to mind some of the old patients he had had for thirty years; some of them come to him with very poor teeth, requiring much attention, he had always instructed them to keep their teeth clean, and use the brush. It was true he had seen some harm cne by using the tooth brush indiscriminately; did not believe in sawing the brush across the face of the teeth, but to brush lengthwise as well, passing the bristles between the teeth. Not alone was the teeth conserved in treatment and attention, but the gums also.

Dr. Samuel R. Steward, Akron, Ohio, thought the treatment of the teeth with the brush should be thorough, employ other means if they would accomplish the desired end. The treatment of the gums was a matter that must be looked at from other standpoint, often requiring other than mechanical treatment. Discussion closed.

Prof. J. M. Carnochan, M. D., New York, then made some extemporaneous remarks upon dentistry, past and present. He said:

The subject of dentistry is one of great importance. The sacred ministers of religion in former times, were supposed to minister to the physical wants of the tribes amongst which they were placed; after a time the surgical art became more or less linked with the mechanism of the barber; in the course of time it assumed a different phase and it now probably stands at the head of all those branches of science which are devoted to the benefit of the human race. Surgery has become divided into a number of departments, and amongst these is dentistry. The art of dentistry now embraces not only the mechanical use of the instruments applied to the teeth, but also a knowledge on the part of the operator of all the vital functions of the system. The mere mechanical dentist at the present day, would be considered a very unfit member of the dental profession. To dentistry, surgery owes one of the greatest boons that science has given to humanity. To Horace Wells, the dentist, of Hartford, is due the discovery of anæsthesia. Dental surgery has done much for general surgery. In the deformity known as hare-lip, for instance the operation of staphylorraphy is now seldom; resorted to, because of the great improvement upon that operation invented by Dr. Sterns, the dentist. The dental art is so associated with the surgery of the jaw, that it is difficult to separate the one from the other. The disease known as *tic douleureux* certainly comes under the head of dental art, and medical men expect to hear of improvements made by dentists in the treatment of this fearful disease.

The dental art has now assumed so important a position, that it must not be excluded from the domain of surgery. It is no longer a mechanical art—it is based upon the science of vitality; and it must and will again become a part of the general profession. There is no sect in medicine but what has been of some advantage to the general art of healing. Dentistry must cease to be an abstraction from the general art.

